# Lipid-based ayurvedic formulations of a single herb-Yashtimadhu (*Glycyrrhiza glabra*): Pharmaceutical standardization, shelf-life estimation and comparative characterization

**DOI:** 10.1016/j.jaim.2023.100711

**Published:** 2023-04-25

**Authors:** Sandeep Chavan, Sushama Bhuvad, Bhagyashri Kumbhalkar, Tanhaji Walunj, Vidya Gupta, Vineeta Deshmukh, Sukumar Sardeshmukh, Sadanand Sardeshmukh

**Affiliations:** a Bharatiya Sanskriti Darshan Trust's Integrated Cancer Treatment and Research Centre, Pune, India; b Atharva Nature Healthcare Pvt Ltd, Pune, India; c Merieux Nutriscience Pvt Ltd, Navi Mumbai, India

**Keywords:** *Glycyrrhiza glabra*, Ayurvedic lipid-based formulations, Ghee and oil, Licorice, Shelf-life, Stability study

## Abstract

**Background:**

Single herb Ayurvedic lipid-based formulations of *Glycyrrhiza glabra* are used as oral, nasal and topical applications for reducing radiotherapy induced side effects in oral cavity cancer patients. These formulations are reported to be de-glycyrrhized, thus minimizing adverse effects of glycyrrhizin on longer consumption. Being a proprietary formulation with specific ratio of herb, lipid and liquid media, there is a need of pharmaceutical standardization and stability study to be conducted for quality control and quality assurance.

**Objective:**

Standardization of *Yashtimadhu Ghrut* (YG) and *Yashtimadhu Taila* (YT) based on pharmaceutical characters, safety tests, chromatographic analysis and stability study.

**Material and methods:**

Two formulations viz., YG and YT were prepared using cow's ghee and sesame oil, respectively. Basic physicochemical analysis, Thin Layer Chromatography, High Pressure Liquid Chromatography of glabridin and 18-β glycyrrhetinic acid, microbial load and heavy metal analysis were performed. Long term (0,3,6,9,12 months) as well as accelerated (0,3,6 months) stability study was conducted and extrapolated shelf-life was calculated for both the drugs.

**Result:**

Organoleptic and basic physicochemical characters were comparable for both the products while safety parameters were within permissible limits. Extrapolated shelf-life was deduced as 1.74 and 0.67 years for YG and YT, respectively.

**Conclusion:**

Single herb- *G. glabra* based lipid formulations were standardized and monographs were established. Shelf-life, though complying with classical Ayurvedic texts, indicates further research work with respect to pre-treatment of lipids and packaging systems for its enhancement.

## 1 Introduction

Ayurveda is based on *Trisutra* viz. causes (*hetu*), symptoms (*linga*) and treatment (*aushadha*) where drugs are used to maintain good health as well as cure diseases [1]. Drugs contribute 25% among the successful treatment parameters (*Chikitsa chatushpada*) and are mainly administered according to the condition of and suitability of the patient [2]. Juice (*swarasa*), paste (*kalka*), decoction (*kashaya*), cold infusion (*hima*) and hot infusion (*phanta*) are the five basic Ayurvedic pharmaceutical forms of a drug (*Panchvidha kashaya kalpana*). However, these basic forms are also used to prepare different specialized formulations viz., lipid-based (*sneha kalpa*), distillates (*arka*), hydro-alcoholic fermentation (*sandhana kalpa*), shakes (*mantha*), treated water (*udaka*), powders (*churna*), tablets-pills (*guti-vati*), wicks (*varti*), concentrates (*rasakriya*), confectioneries (*khanda*), dispersed metallic preparations (*ayaskriti*), starches (*sattva*), alkaline salts (*kshara*), etc.; as per the pathology [1].

Among these preparations, medicated lipids (*Sneha Kalpana*) in Ayurveda can be considered a Lipid Based Drug Delivery System (LBDDS) since the pharmaceutical process of preparing oleaginous formulations uses basic dosage forms of herbs like paste, juice, decoction and other liquid media like milk, water, butter milk, etc.; in specific proportion by subjecting heat (*sneha paka*) till the water portion get completely evaporated. This facilitates uniform distribution and absorption of fat and water-soluble chemical constituents. The end stage of production is specific for oils and ghee (*paka siddhi)* and tested to determine complete water evaporation by heating the residual material on flame wherein no crackling sound is audible. Moreover, *Murchhana*/fortification of lipids with certain herbs is also suggested to alter solubility and absorption ability; remove moisture content related to rancidity (*amadosha*) and bad odour while additionally imparting appealing colour and odour to oil or ghee [3].

The active bioactive compounds such transferred into the lipid solvents like sesame oil and cow's ghee are used for oral administration (*paan*), nasal instillation (*nasya*), dermal route (*abhyanga*), per rectum (*enema*), per vaginum (*uttarbasti*) and topical administration [4]. Cell membranes are primarily composed of bimolecular lipid matrix which determines membrane permeability characteristics with specificity of drug molecules. Lipid-based drugs are easily absorbable as they diffuse more rapidly across the cell membrane by passive diffusion [5]. Recently, LBDDS has shown effective size dependent properties since poorly water-soluble drugs pose challenge with regards to its solubility and bioavailability. This system is superior because of obvious advantage of higher degree of biocompatibility and versatility. Lipid formulations in Ayurveda are diverse from single to polyherbal; single lipid to combination of lipids; with and without milk; single or multiple cycles of drug enrichment (*avartana*), etc. Such variations help to meet a wide range of product requirements as per the disease condition, route of drug administration, cost of product, stability, safety and efficacy [6].

Interesting reports of such lipid-based formulations in several chronic and deadly diseases are available. One of the reports has studied single herb *Yashtimadhu/Glycyrrhiza glabra* L. (GG) lipid-based (sesame oil and cow's ghee) formulations in oral cavity cancer patients for inflammatory conditions like stomatitis, mucositis, wound healing, skin discoloration, burning sensation, hyperacidity, etc. induced due to chemotherapy and radiotherapy [7]. Though GG is a popular, economically important, and broadly used herb in clinical practice [8], therapeutic efficacy depends upon the quality and quantity of raw material and its active chemical constituents in the finished product. Previous report proved that GG lipid-based formulations are de-glycyrrhized and highlighted that only selective phytocompounds get dissolved/dispersed in the lipids [9].

Overall, GG has been ascribed to 22 dosage forms and 252 formulations. Among them, 179 and 62 are administered through internal and external routes, respectively [10]. GG has sweet (*madhura)* and bitter *(tikta)* tastes, sweet effect after bio-transformation (*madhura vipaka*) and cold potency (*sheeta veerya*). It is heavy to digest (*guru*), unctuous (*snigdha*), spermatogenic (*shukrala*) and possesses hair texture improving (*keshya*), voice quality improving (*swarya*), strengthening (*balya*), complexion enhancing (*varnya*), eye tonic (*chakshushya*) and *Vata-Pitta Dosha* alleviating properties. It is recommended in several disease conditions like oedema (*shotha)*, debility (*kshaya*), gout (*vatarakta*), wound (*vrana*), erysipelas (*visarpa*), etc [11].

Classically, such lipid formulations have been attributed with very less shelf life [12]. This is probably due to rancidity [13]. Many Ayurvedic pharmaceutical companies manufacture thousands of such lipid formulations for Ayurvedic practitioners; however, standardization and characterisation are not well reported. The actual transformations during lipid formulations are seldom studied. Recent guidelines have further emphasized on stability study of Ayurvedic formulations for the assessment of bioactive components, safety aspects and identification of proper storage conditions during the ageing process [14]. Thus, there is a need for quality control and quality assessment of raw materials as well as pharmaceutical preparation process to get batch to batch consistency. Though general rules are laid for manufacturing of lipid formulations wherein ratios of herbs, lipids and liquid media are reported [12]; the present study was designed to pharmaceutically standardize proprietary formulations of GG; viz., *Yashtimadhu Ghrut* (YG) and *Yashtimadhu Taila* (YT) having animal fat (*ghrut*) and vegetable oil (*taila*) as base, respectively. Basic physicochemical, chromatographic and safety parameters were tested for both. The standardized and characterized formulations were further subjected to stability study and extrapolated shelf-life was calculated. The developed monograph and shelf-life study will probe for new research in the assessment of such single herb processed lipid-based formulations.

## 2 Material and methods

### 2.1 Raw materials

The crude drug GG as stolon/stem was purchased from Manakarnika Aushadhalay, Pune, India and authenticated from Indian Drug Research Association and Laboratory, Pune by following standards as per Ayurvedic Pharmacopoeia of India (API). Voucher specimen was deposited at the institutional repository (V-29). Cow's ghee (CG) was purchased from B.V. Chitale Group, Pune, India while sesame oil (SO) from Ramkrishna Oil Mills, Pune, India.

### 2.2 Instruments, standards and reagents

The temperatures were monitored by a digital infrared thermometer, Non-contact Liquid Crystal Display Laser meter gun style (range-room temperature to 1000 °C, accuracy- ±3 °C, Gain Express Holding Ltd, Hong-Kong) during the manufacturing process. High-Pressure Liquid Chromatography (HPLC) analysis was performed on Thermo Scientific Dionex Ultimate 3000 UHPLC Rapid Separation system (Waltham, USA). Heavy metal testing was done on Inductively Coupled Plasma Optical Emission Spectrophotometry (ICPOES, iCAP7200 Duo, Thermo Fisher Scientific, Waltham, MA, USA) after closed acid digestion of samples in Ethos Easy Advanced Microwave Digestion System (Milestone SRL, Milan, Italy). Stability chambers (Thermolab Scientific Equipment Pvt. Ltd., Palghar, India; Equipment 1 Serial no. 522/12/09–10 and Equipment 2 Serial no.521/12/09–10 with a range of temperature 20–60 °C and RH 40–98% RH) were used for accelerated and long-term storage of drugs, respectively. Phytochemical Reference Standards Glabridin (purity ≥98%) and 18β-glycyrrhetinic acid (purity ≥97%) were purchased from Sigma–Aldrich, India; HPLC grade methanol and acetonitrile from High Purity Laboratory Chemical Pvt. Ltd. (Mumbai, India) while analytical grade O-Phosphoric acid from Thomas Baker (Mumbai, India). Elemental Standards for As, Cd and Pb were purchased from Merck (Certipur, Darmstadt, Hesse, Germany) and of Hg from Sigma Aldrich (TraceCERT, St. Louis, MO, USA).

### 2.3 Preparation of formulations

YG and YT were prepared using standard procedure [15], as commercial triplicate batches in 3 days at Atharva Nature Healthcare Pvt. Ltd., Pune, India with the following modifications.

#### 2.3.1 Preparation of decoction

One part of coarse powder of GG was soaked in 16 parts of water (w/v) for 18 h in Stainless Steel (SS) vessels. Next day, it was boiled to reduce to 3/4th part (v/v) on medium flame (90–100 °C) and then filtered through mesh #40 in hot condition.

#### 2.3.2 Preparation of medicated lipids

The prepared decoction was mixed with equal parts of CG (v/w) or SO (v/v) in tin coated brass vessels (for YG- 70 L capacity, diameter 55 cm, height 30 cm; for YT- 20 L capacity vessel, diameter 35 cm, height 20 cm) for preparing YG and YT, respectively. The mixture was continuously stirred while heating at 90–100 °C in initial phase and up to 110 °C during the final phase of preparation, till water got evaporated wherein temperature was taken every 15 min for YG and 5 min for YT. The end point of finished product was ascertained by classical signs of *sneha siddhi* [12] and *madhyam paka* [12] was achieved in both the products. Finally, YG and YT in hot condition were filtered through muslin cloth in SS vessel and self-cooled.

#### 2.3.3 Storage and packaging for stability study

Finished product YG, 140 g, was packed in wide mouth white-coloured High-Density Polyethylene (HDPE) jar (GC100, V-PLA Products, Pune, India) with 10% space left, sealed with aluminium foil (65 mm, Liddo Brand, BS Foils Pvt Ltd, Palwal, India), capped, labelled and shrunk with a transparent plastic sheet (8 jars per batch). YT, 100 mL, was filled in 110 mL capacity amber-coloured Polyethylene Terephthalate (PET) bottle (Sheth PET and Polymers Pvt Ltd, Wada, India), closed with aluminium cap (25 mm, Quality Closures Pvt Ltd, Baramati, India), threaded, labelled and shrunk with transparent plastic sheet (8 bottles per batch).

### 2.4 Basic physicochemical analysis

Raw material, intermediate and finished product, each for YG and YT, were tested. Organoleptic characters such as odour, taste, appearance and colour were observed [16]. Physicochemical parameters like loss on drying (LOD), specific gravity, weight per ml, refractive index, congealing point, sieve analysis, bulk and tapped densities, water soluble extractive, alcohol soluble extractive, ash value, acid insoluble value, saponification value, iodine value, acid value, peroxide value, un-saponifiable matter and Reichert Meissel (RM) value was determined as per guidelines [14].

### 2.5 Chromatographic analysis

Ethanolic extract of raw drug and methanolic extract of finished products were run on pre-coated Silica Gel G60F_254_ plate in mobile phase Toluene: ethyl acetate: formic acid: (5:4:1), under standard Thin Layer Chromatography (TLC) conditions [14] and chromatograms were recorded under Ultra-Violet (UV) radiation at short UV (254 nm) and long UV (366 nm) wavelengths as well as after spraying anisaldehyde sulphuric acid reagent. Bioactive components glabridin and 18-β glycyrrhetinic acid were identified and quantified in YG and YT using HPLC as reported previously [9]. Both the lipid formulations were extracted using a binary solvent system of methanol and *n*-hexane. Separation was performed on Hypersil gold column (Thermofisher Scientific, Waltham, USA) maintained at 40 °C using 0.2% ortho-phosphoric acid with pH 3.5 in water (eluent A) and acetonitrile (eluent B) as a binary gradient mobile phase. The gradient program was set as 70:30 (0–2 min), 20:80 (7–12 min), and 70:30 (15–16 min) wherein from 2 to 7 min interval, the concentration of organic mobile phase B was gradually increased from 30 to 80 and from 12 to 15 min interval, it was gradually decreased from 30 to 80. The flow rate of 0.7 mL/min was maintained throughout the analysis. Glabridin and 18β-glycyrrhetinic acid were detected at wavelengths 230 and 254 nm, respectively. HPLC was performed for all the stability study batches at all the time points. Validation of the HPLC method based on linearity, range, specificity, accuracy and precision for these compounds has been detailed in the previous report by the authors [9].

### 2.6 Safety parameters

Microbial load, fungal load and *Escherichia coli* as specific pathogen was assessed using standard protocol [14]. Heavy metals (Hg, As, Cd, Pb) were tested by placing 100 mg sample, 6 mL conc. HNO_3_ and 2 mL H_2_O_2_ in the microwave vessel, were allowed to react and then digested gradually for 15 min to achieve 200 °C temperature, then maintained for 20 min and finally cooled to room temperature. Standard solutions of 1000 mg/L were diluted to 5, 10, 20 and 50 μg/L and digested samples were made up to 50 mL using de-ionized water. The final samples were aspirated in Argon gas flame in the ICPOES system with an auxiliary gas flow of 0.5 L/min. The intensities were measured at wavelengths 184.959, 194.227 and 253.652 for Hg, 189.042, 193.759 and 449.423 for As, 228.802 and 226.502 for Cd while 220.353 and 216.999 for Pb in axial view. The wavelengths exhibiting *R*^*2*^ value of more than 0.999 were selected for calculating the respective elements. The calibration graph for standards was plotted using Qtegra Software Version 2.6 (Thermo Fisher Scientific, Waltham, MA, USA) and the final concentration was measured in ppm.

### 2.7 Stability testing and shelf-life determination

The study was conducted as per the International Council for Harmonisation (ICH) of Technical Requirements for Pharmaceuticals for Human Use guidelines Q1A (R2) for evaluation of the shelf life of YG and YT [17]. Eight samples were stored and maintained at 40 ± 2 °C, RH 75 ± 5% for an accelerated study (AS) of 6 months and at 30 ± 2 °C, RH 65 ± 5% for long term (LT) study of 12 months. The variables were tested on Day 0 and after every 3 months. Acid value, peroxide value, iodine value, unsaponifiable matter, moisture content and HPLC were studied for both, YG and YT, while RM value and congealing point additionally for YG, were used for calculating the shelf life. Moreover, microbial load, fungal load, presence of *E. coli* along with specific gravity and refractive index were also tested. The evaluation of degradation during storage was studied and 10% changes in properties of the samples were set to extrapolate the stability data. Aging factor 5 and 3.3 was used to determine shelf-life for climatic zone I & II and III & IV countries, respectively. The months when 10% degradation occurs were calculated by the expression [17] as given below:

Numberofmonthswhen10%degradationoccurs=[0monthassayvalue−{0monthassayvalueX10/100}]−Intercept/Slope

### 2.8 Statistical analysis

The obtained data in physicochemical parameters and microbial load were analysed by calculating mean ± standard error of mean using Microsoft excel 2010. Data acquisition and integration of HPLC were controlled by Thermo Scientific Chromeleon 7.2 chromatography data system software.

## 3 Result and discussion

Being a proprietary medicine, monograph for YG and YT, each, was developed and found to be in compliance with API standards. The details are mentioned in the supplementary file (supplementary file 1, Table S1). In the process of lipid formulations, sesame oil or cow's ghee was mixed directly with YT decoction without any prior treatment (*murchhana*) since therapeutic effect of the single herb medicated ghee/oil is clinically reported [18]. Though *murchhana* is one of the important and recommended steps, it has been shown that herbs used in *murchhana* process do not impart much effect against oxidative damage in lipids occurring at high temperatures during manufacturing process [19].

### 3.1 Raw and in-process pharmaceutical standardization

#### 3.1.1 Raw material analysis

GG consisted of dried stolon pieces with brown outer layer, internally yellowish brown, longitudinally wrinkled, occasionally having small buds, smooth transversely, coarsely fibrous, with characteristic odour and sweet in taste. CG was thick semisolid mass, with characteristic odour, sweet in taste and yellow in colour while SO was viscous liquid with characteristic odour; sweet and astringent taste and dark yellow colour.

LOD of GG was within the permissible limit (6.41 ± 0.45 %w/w) ensuring a poor environment for growth of moulds and bacteria for sustaining quality of the raw material. Ash value (4.85 ± 0.22 %w/w) was within limits denoting the presence of natural essential elements, absence of adulteration with any inorganic material and proper harvesting age. Acid insoluble ash (0.62 ± 0.36 %w/w) within the normal range indicated cleanliness of the plant material with respect to extrinsic silicates. Alcohol and water-soluble extractive values (4.64 ± 0.11, 35.08 ± 6.39 %w/w, respectively) represented solubility of phytocompounds in a particular solvent [20]. All the basic physicochemical characters of GG complied with API, thus demonstrating its authenticity.

#### 3.1.2 In-process analysis

Uniform particle size distribution is required for uniform dosage formation. GG decoction was prepared from coarse particle sized GG powder passing completely through sieve #10 (1.7 mm mesh). The percentage of total dissociated solid content was found to be less in decoction prepared with fine powder as compared to that of coarse powder [21] since uniform extraction probably occurred during heating. In this study, more than 63, 72, 75 and 80% of coarse GG was retained in sieves #40, # 60, #80, and #100 respectively, revealing 40% particles between 1.7 mm and 350 μm; 40% between 350 and 150 μm and 20% above 150 μm. Further, bulk density of this coarse GG powder ranged between 0.31 and 0.36 and tapped density between 0.46 and 0.38 g/cc which indicated its coarse and fibrous nature. GG decoction was a thin clear liquid with sweet taste, yellowish brown colour and characteristic odour. It exhibited pH of 5.39 ± 0.06 and specific gravity of 1.02 ± 0.002 in YG batches while pH 5.48 ± 0.23 and specific gravity 1.02 ± 0.001 in YT batches. Low pH value denoted acidic nature of GG decoction while specific gravity indicated dense liquid due to soluble matter.

#### 3.1.3 Ratio of decoction to lipids

In the present study, both the lipids were added with decoction in equal proportion instead of 1:4 ratio (1 part oil v/v or ghee w/v: 4 parts decoction) as reported previously [12]. Pilot experiments of 1:4 ratio revealed that GG itself being sticky, when heated with oleaginous substance created difficulty in completing the process till the end stage (*pakasiddhi)*. The decoction residue easily got caramelised/carbonised (*kharapaka*) which is not expected for multipurpose therapeutic activity [12].

#### 3.1.4 Pharmaceutical yield

All the YG and YT batches had optimal yield with minimal loss and the details are given in (Table 1).

**Table 1:** Pharmaceutical yield of *Yashtimadhu Ghrut* (YG) and *Yashtimadhu Taila* (YT).

Batch	Quantity of GG	Quantity of ghee/oil	Yield	Loss	% Loss
	Kg	Kg	Kg	g	w/w
YG1	10	40	39.6	400	1
YG2	8	32	31.45	550	1.72
YG3	8	32	31.5	500	1.56
Mean ± SD	-	-	-	483.3 ± 76.4	1.43 ± 0.4
	Kg	L	L	mL	v/v
YT1	0.625	2.5	2.25	250	11.11
YT2	0.625	2.5	2.4	100	4.16
YT3	0.625	2.5	2.4	100	4.16
Mean ± SD	-	-	-	150.0 ± 86.6	6.48 ± 4.0

### 3.2 Analysis and characterisation of finished products

#### 3.2.1 Ayurvedic analysis for end point in manufacturing process of lipid formulations

The finished product was ascertained by appearance of froth (*phenodgama*) in YT, disappearance of froth (*phenashanti*) in YG, rolling the decoction residue between the finger to make a wick *(vartipariksha*) and lightning the wick of ghee/oil so that no cracking sound was heard (*shabdahinata*). These tests confirmed evaporation of moisture completely and expected heating of oil/ghee at the end of the process. YG and YT, both were thick viscous liquid, yellow in colour and with characteristic odour.

#### 3.2.2 Comparative physicochemical analysis of lipids in raw and finished product form

The analytical results of the physicochemical parameters of cow's ghee and sesame oil along with their finished product counterparts are given in Table 2.

**Table 2:** Physicochemical analysis of lipids.

Parameter	Unit	Raw materials		Finished products	
		CG	SO	YG	YT
Specific gravity	-	0.92 ± 0.002	0.92 ± 0.002	0.91 ± 0.0005	0.92 ± 0.002
Loss on drying at 105 °C	%	0.20 ± 0.07	0.14 ± 0.07	0.32 ± 0.14	0.08 ± 0.001
Acid value	-	1.09 ± 0.16	1.83 ± 0.11	1.47 ± 0.06	1.63 ± 0.20
Refractive Index	-	1.53 ± 0.001	1.54 ± 0.002	1.54 ± 0.0002	1.55 ± 0.001
Saponification value	-	181.35 ± 32.2	153.65 ± 16.6	223.13 ± 8.1	210.68 ± 15.95
Iodine value	-	43.19 ± 7.56	102.13 ± 20.4	29.75 ± 1.94	72.47 ± 20.77
Peroxide value	-	3.26 ± 1.55	2.06 ± 0.58	1.33 ± 0.37	0.57 ± 0.29
Unsaponifiable matter	%	4.63 ± 2.28	4.25 ± 2.63	0.77 ± 0.31	0.75 ± 0.37
Reichert Meissl value	-	37.41 ± 7.64	NA	35.92 ± 2.08	NA
Presence of cotton seed oil	-	NA	Absent	NA	Absent
Congealing point	°C	24.0 ± 3.0	NA	25.0 ± 3.0	NA

Data: Mean ± SEM, *n* = 3; CG- Cow's ghee, SO- Sesame Oil, YG- *Yashtimadhu Ghrut*, YT- *Yashtimadhu Taila*.

LOD in these lipids was between 0.1 and 0.25% which complies with permissible limit in lipid-based products [22]. Higher moisture content is responsible for rancidification consisting chemical processes of generating highly reactive molecules in rancid foods and oils, producing unpleasant odour and taste [23]. This may also destroy active constituents of such lipid-based formulations. Rancidification can be determined by acid value as well as free fatty acids value. These acid values of cow's ghee, sesame oil, YG and YT ranged between 1 and 2, thus indicated least rancidity.

It is possible that the ratio of fatty acids and their derivatives in raw ghee/oil and their processed counterparts YG/YT may alter due to adulteration/impurities, effect of temperature and inclusion of herbal drug during preparation, respectively. These changes can be assessed by measuring refractive index. The raw lipids complied with the standards while the refractive indices of both YG and YT processed with GG showed only slight change by 0.01 when compared with their respective raw lipids.

Saponification value measures the average molecular weight of all the fatty acids present in direct proportion to lipids. YG showed slight increase while YT exhibited considerably higher saponification value as compared to their respective lipid bases, indicating low-molecular weight fatty acids formed probably due to heating [24]. Similarly, higher iodine value determining the amount of unsaturated fatty acids, decreases stability of the lipids. Both, YG and YT depicted lower iodine values as compared to their counterparts, CG and SO, indicating presence of less unsaturated fats. Further, the peroxide value, which measures rancidity caused in unsaturated fat or oil due to auto-oxidation, helps to assess spoilage of the product [25]. In both, YG and YT, peroxide value were lower as compared with that of CG and SO, respectively; indicating minimum rancidity. Detail fatty acid analysis using GC–MS may throw light on this aspect.

Quantification of unsaponifiable or non-fatty matter soluble in the fats is used to determine any such plant constituent being transferred to the medicated lipids during processing. For both, YG and YT, the unsaponifiable matter decreased considerably (∼3.5%) which can be attributed to the procedure of heating and/or separation of residue left at the endpoint.

RM value is useful to determine the low-carbon fatty acids of ghee like butyric (4:0) and caproic (6:0), thus qualifying purity or adulteration of fats. RM value of both YG and CG (cow's ghee 24–28) did not show any considerable difference and complied with the standard requirements [26]. The congealing point of a liquid or of a melted solid is the highest temperature at which it solidifies. Both, YG and CG had similar congealing point. Thus, it can be stated that RM value and congealing points were not affected by heating CG with decoction of GG.

#### 3.2.3 Chromatographic analysis

The spots detected in TLC are detailed in Table 3. GG showed 16, 21 and 18; GG decoction exhibited 12, 14 and 10; YG had 1, 4 and 11 while YT showed 13, 10 and 14 number of spots at 256 nm, 366 nm and after derivatization, respectively. Decoction and finished products shared some common *R*_*f*_ values with raw material indicating the existence of common phytoconstituents in them, such as 0.52, 0.78 and 0.91 at 366 nm and 0.96 after derivatization, in GG decoction; 0.59 and 0.71 at 256 nm; 0.17, 0.52 and 0.68 at 366 nm as well as 0.13 and 0.69 after derivatization, in YG; 0.69, 0.80 and 0.95 after derivatization while in YT; 0.62, 0.67, 0.71, 0.72 and 0.86 at 256 nm; 0.43, 0.52, 0.62, 0.63, 0.69 and 0.71 at 366 nm and 0.29, 0.67 and 0.96 after derivatization. Interestingly, low range *R*_*f*_ values observed in the raw materials disappeared in the finished products i.e., 0.03, 0.07, 0.13, 0.17, 0.29 and 0.34. This may be due to aqueous-lipid separation process or due to transformation into other compounds because of heating [27].

**Table 3:** Thin Layer Chromatography profile of raw material, intermediate and finished products.

Sample	*R*_*f*_ value		
	256 nm	366 nm	After derivatization
GG	16 spots: 0.03, 0.07, 0.13 (B), 0.42, 0.53 (Br), 0.59, 0.62, 0.66, 0.67, 0.71, 0.72, 0.78, 0.80, 0.84, 0.86, 0.91 (B)	21 spots: 0.03 (B), 0.11(R), 0.17, 0.28, 0.43 (Y), 0.48, 0.52 (Y), 0.53, 0.58 (Flu B), 0.62, 0.63(B), 0.68, 0.69, 0.70(R), 0.71(R), 0.78, 0.79(Y), 0.80(B), 0.84, 0.86 (R), 0.91(B)	18 spots: 0.03(Bl), 0.07, 0.13 (Y), 0.29, 0.34(B), 0.51(V), 0.52(B), 0.61(PV), 0.62, 0.67, 0.69, 0.78(R), 0.80 (Gy), 0.84, 0.86 (B), 0.91 (B), 0.95 (Gr), 0.96(R)
GG decoction	12 Spots: 0.08, 0.24, 0.26, 0.29, 0.38, 0.44, 0.47, 0.59, 0.65, 0.71 (B), 0.76, 0.79 (Gr)	14 spots: 0.04, 0.13, 0.17 (Y), 0.29, 0.33, 0.34(Y), 0.40, 0.47 (B), 0.51, 0.52, 0.59 (Flu B), 0.68, 0.80, 0.97 (Y)	10 spots: 0.04, 0.06, 0.09 (Gr), 0.13, 0.15 (B), 0.40, 0.43 (PV), 0.64, 0.65, 0.69 (Y)
YG	1 spot: 0.95(B)	4 spots: 0.49, 0.59, 0.57 (LB), 0.64(R)	11 spots: 0.54, 0.57(B), 0.59(R), 0.66, 0.69, 0.70, 0.75, 0.79, 0.80, 0.82, 0.95 (B)
YT	13 spots: 0.48, 0.62, 0.63, 0.65 (B), 0.67, 0.68 (Gr), 0.69(Y), 0.71 (Gr), 0.72, 0.77, 0.82, 0.86, 0.94 (B)	10 spots: 0.43, 0.52, 0.56 (B), 0.62(B), 0.63, 0.67, 0.69, 0.71, 0.76(R), 0.77 (B)	14 spots: 0.27, 0.29(B), 0.43 (P), 0.55 (Gr), 0.57(V), 0.60 (O), 0.67, 0.71 (B), 0.72 (Y), 0.76(PV), 0.77(P), 0.81(B), 0.87, 0.96 (Br)

B = Blue, Br = Brown, R = Red, Gr = Green, Gy = Grey, V = Violet, PV = Pink Violet, Bl = Black, Flu B = Fluorescent blue, Y = Yellow, P = Pink, O = Orange, LB = Light blue.

HPLC analysis of three compounds viz., glabridin, glycyrrhizin and 18-β glycyrrhetinic acid has been reported at different steps of pharmaceutical preparation; i.e., raw material, decoction, decoction residue, YG, YT, and product residue. YG contained 95 and 112.3 ppm while YT contained 138 and 500 ppm of 18-β glycyrrhetinic acid and glabridin, respectively. Interestingly, these results indicated total de-glycyrrhized YG and YT while their residues showed presence of glycyrrhizin [9]. As reported previously, glabridin and 18-β glycyrrhetinic acid identified in these lipid formulations can be considered as identification and quality control markers since these two metabolites provide anti-cancer, anti-inflammatory, anti-mutagenetic, anti-proliferative, antioxidant [9] and radioprotective activities [19].

### 3.3 Safety parameters

Microbial load was estimated and found to be within permissible limit which represents preparation and storage of intermediates and products in hygienic conditions and their aseptic handling. Similarly, heavy metal assessment using ICPOES was done to check for any contamination thereby avoiding hazards on the body. In both YG and YT, heavy metals were within permissible limit, denoting its safe use in the human body (Supplementary file, Table S2, S3).

### 3.4 Stability study and shelf-life determination

Stability testing is a reliable way to ensure the effectiveness of a new product with respect to physicochemical properties, phytochemicals and contaminants over the time period under the influence of environmental factors such as temperature, humidity and light. It also facilitates shelf-life calculation in recommended storage conditions. The formulation is considered to be stable if ‘no significant change’ occurs during any time of testing at accelerated or real time storage conditions [14].

**Fig. 1:**
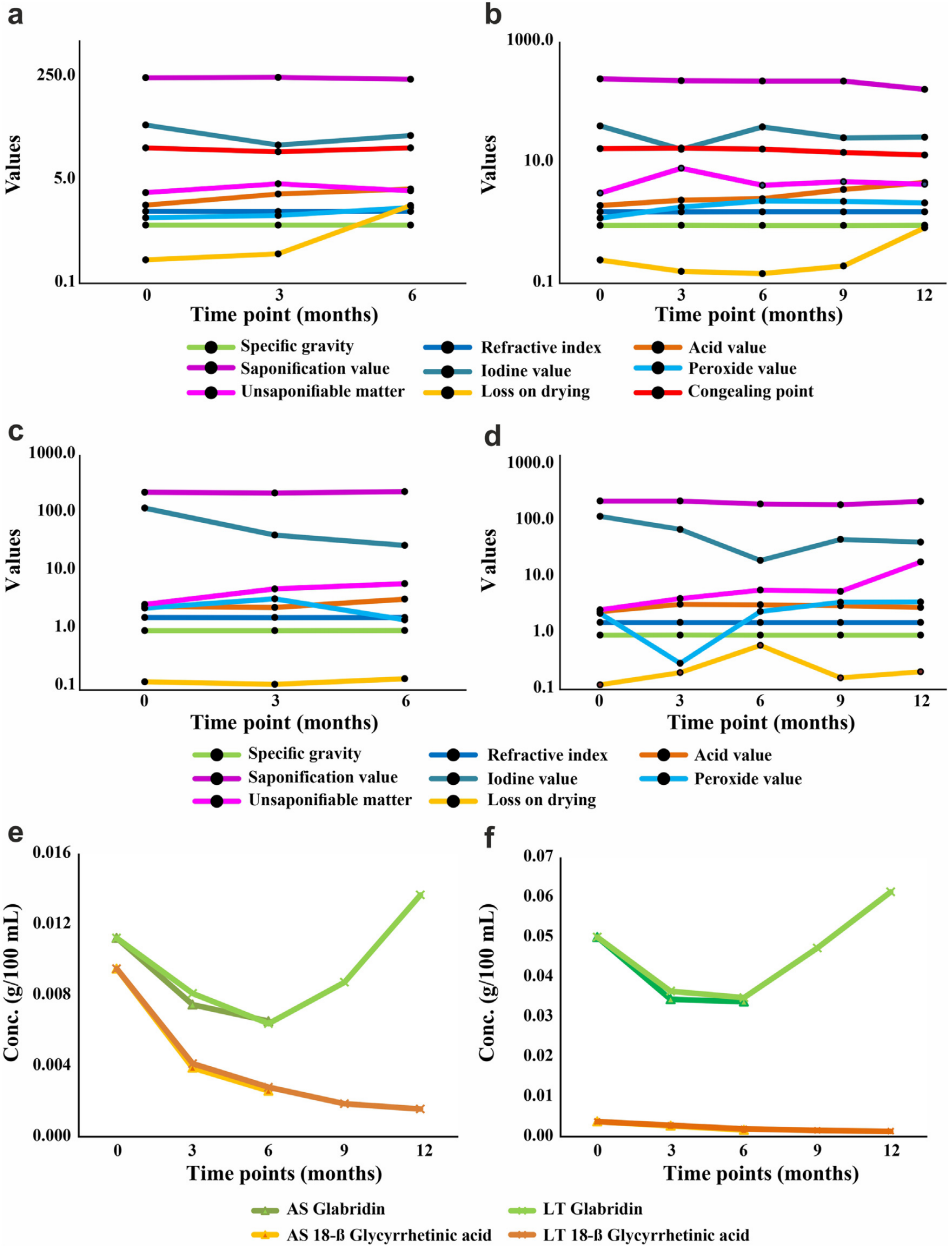
Stability study of *Yashtimadhu Ghruta* (YG) and *Yashtimadhu Taila* (YT) (a) Accelerated study (AS) of YG (b) Long term (LT) study of YG (c) AS of YT (d) LT study of YT (e) Evaluation of phytocompounds in YG under AS and LT study (f) Evaluation of phytocompounds in YT under AS and LT study.

On the basis of data generated in this study, in accelerated and long-term storage conditions of YG; refractive index, specific gravity and congealing point exhibited no significant change. Acid, peroxide and unsaponifiable values gradually increased while iodine and saponification values decreased. LOD decreased gradually with a sudden increase at the 12th month. Bioactive compounds i.e., glabridin and 18-β glycyrrhetinic acid exhibited gradual decrease over the period of 6 months. In case of glabridin, the values increased in the 9th and the 12th month as compared to the initial value (Fig. 1, supplementary file, Table S4, Fig. S1, S2, S3 and S4).

In accelerated condition of YT; acid and saponification values decreased initially and increased at the 6th month. Unsaponifiable matter and LOD exhibited gradual increase while iodine value and 18-β glycyrrhetinic acid showed gradual decrease but glabridin content increased similar to YG. Peroxide value increased till the 3rd month and then decreased at the 6th month. Specific gravity and refractive index were stable till the 6th month. In case of long-term storage condition; acid, peroxide, unsaponifiable and LOD values increased while saponification and iodine values decreased as compared to initial value (Fig. 1, supplementary file, Table S5).

Overall, in accelerated condition of YG; physicochemical characters like saponification value, unsaponifiable matter, congealing point, specific gravity, and refractive index varied up to ±15% while acid, peroxide, iodine and LOD values altered beyond ±25% from initial value. Bioactive compounds, glabridin and 18-β glycyrrhetinic acid differed up to 25% from the initial value. However, physicochemical characters in accelerated condition of YT; saponification value and LOD degraded up to ± 15% while acid, iodine, peroxide and unsaponifiable values along with bioactive compounds changed beyond 25% from the initial value (Supplementary file, Table S8, S9).

Acid value indicates the number of carboxylic acid groups in fatty acid compounds. In rancidity of oil/fat over the period of time; triglycerides get converted into fatty acid and glycerol, causing increase in amount of acid. In the present study, it can be considered that catalytic effect may have occurred due to transformation of triglycerides into free fatty acids in YG. Extent of lipid peroxidation designated by peroxide value indicates initial incidence of rancidity in unsaturated fats and oils. Free unsaturated acids are oxidized more easily and quickly thus higher acid value indicates high peroxide value. Though there was an increase in the peroxide value, signifying chances of developing rancidity over the period of 6 months, it was within the permissible limit of un-rancidification [28].

The iodine value specifies quantity of iodine absorbed at unsaturation which expresses the amount of unsaturation in a fat. A large fall in iodine value over 3 months with a rise thereafter but below the initial level in the present study might be corroborated with more oxidation due to high unsaturation in YG. Further, loss on drying measuring the moisture content of the product is an important factor in preservation and resistance to deterioration. The amount of water content can affect texture, taste, appearance and stability of a formulation. In case of YG, LOD was within permissible limit till the 9th month, but exhibited sharp rise at the 12th month. However, in YT, LOD comparatively remained stable till the 6th month. Higher moisture content at later stages of stability study might be due to the packaging style used for these two products (Supplementary file, Table S4, S5).

Interestingly, the safety parameter of microbial count, fungal count and *E. coli* did not show any significant alteration over the study period in both, YG and YT. Similarly, the heavy metals also remained stable (Supplementary file, Table S4, S5). This ensured the quality of container closure system used in this study and could be considered sufficient to maintain the environment necessary for preservation of the products.

In TLC analysis of YG, there was a shift in the range of *R*_*f*_ values from higher to lower side at 254 and 366 nm over the study period, i.e., from 0.790 to 0.516, 0.991 to 0.851. Higher *R*_*f*_ values disappeared with generation of new spots at lower side. In YT, *R*_*f*_ values were stable at 256 and 366 nm with generation of new spots (Supplementary file, Table S6, S7). However, HPLC analysis for bioactive compounds revealed gradual decrease in glabridin, 18-β glycyrrhetinic acid over 6 months in both, YG and YT, however these proportions were higher in YT as compared to YG (Supplementary file, Table S4, S5).

Extrapolated shelf-life of YG and YT was calculated with 10% degradation rate from the physicochemical characters and their bioactive compounds at accelerated storage condition. The extrapolated shelf-life of YG and YT were 1.74 years and 0.67 years for climatic zones I & II, respectively; while for climatic zones III & IV, the extrapolated shelf life of YG and YT were 2.63 years and 1.01 years, respectively (Table 4).

**Table 4:** Calculation of shelf life of YG and YT using accelerated stability study data.

	YG		YT	
	10% degradation	Months when 10% degradation occurs	10% degradation	Months when 10% degradation occurs
Acid value	1.75	0.82	1.98	0.56
Peroxide value	1.09	0.95	103.57	0.13
Iodine value	36.48	1.43	2.24	9.59
Unsaponifiable matter (%)	2.81	21.64	1.49	2.15
Loss on drying at 105°C (%)	0.22	0.94	0.06	2.55
Congealing point (°C)	15.30	4.27	NA	NA
Glabridin (g/100 mL)	0.01	19.95	0.046	0.95
18-ß Glycyrrhetinic acid (g/100 mL)	0.01	0.50	0.003	1.10
Mean months		6.31		2.43
Climatic zone I and II		20.83 (1.74 years)		8.03 (0.67 years)
Climatic zone III and IV		31.57 (2.63 years)		12.16 (1.01 years)

The shelf-life of the various dosage forms including ghee and oil has been reported to be 4 months by *Acharya Sharangadhar* in the medieval period [12]. While a contradictory report by *Acharya Bhavmishra*, quoted that medicated ghee loses its potency after 1 year and oil (medicated or plain) gains more potential as it grows old [11]. Moreover, *Acharya Charaka* in *Unmadchikista Adhyaya* has explained ten-year-old ghee (*Purana*) and 100-year-old ghee (*Prapurana*) attributing pungent-bitter taste, strong irritant odour and cold potency. It has lac juice like appearance, alleviates all the three doshas viz., Vata, Pitta, Kapha and useful in insanity (*unmada*), epilepsy (*apasmara*) and demoniac seizures/extra-terrestrial effects (*grahadosha*) [1]. As such there are no clear methods stated regarding longer shelf-life of ghee and there is a scope for future study. Currently, the Ministry of AYUSH, Government of India has published the shelf-life of medicated ghee (*Ghruta*) and oil (*Taila*) as 2 and 3 years, respectively [29] while, The Ayurvedic Pharmacopeia of India has mentioned 16 months shelf-life of medicated ghee/oils if preserved in glass, steel or polythene container [30].

Currently, various synthetic or natural antioxidants like punicalagin [31], lipase enzymes [32], etc. are commercially used to increase shelf-life of *ghruta* while partial or complete hydrogenation [33] is carried out to increase the shelf-life of vegetable oil. Along with it, specific packaging materials (rigid container, semi-rigid packages, flexible pouches) are used to prevent deterioration from oxygen, humidity, light, temperature, tainting and traces of metals. Use of such synthetic antioxidants or partial hydrogenation processes to enhance the shelf-life of products can cause health hazards. The partial hydrogenation leads to production of trans fats. It may have deleterious effect on human health as it increases Low Density Lipoproteins and decreases High Density Lipoprotein also, thus increasing the risk of cardiovascular diseases [34]. Hence, recently, herbal antioxidants are preferably used to increase shelf-life of oil/ghee with comparable results to synthetic antioxidants. These natural antioxidants present in the herbs help to maintain oxidative stability of ghee/oil. Similarly, detailed description of “*Ghruta/Taila murchhana”* which is carried out by heating lipids with specific herbs before using them to develop a therapeutic formulation has also been introduced by *Govindadas Sen* in *Bhaishajya Ratnavali*. It reduces the impurities (*amadosha*) and enhances the odour/flavour of the oil/ghee [3]. The herbs used in this process have their own antioxidants and therapeutic value, hence they may improve the chemical and oxidative stability along with additions of therapeutic properties of the lipid-based formulations. However, *murchhana* was not done in the present study to avoid interference in the physicochemical and chromatographic analysis of such single herb-based lipid formulations. However, it highlights further scope of studies to be undertaken to enhance shelf-life of lipid-based Ayurvedic formulations.

## 4 Conclusion

Ayurvedic proprietary formulations, YG and YT both, were standardised based on physicochemical characters, chromatographic analysis and safety parameters while the stability study derived the shelf-life. The effect of *murchhana* as well as other packaging materials can be studied further to improve the shelf-life. Pre-clinical and clinical assessments for several ailments can also be carried out to evaluate their therapeutic potential with the estimated bioactive compounds.

### Source of funding

This research was funded by 10.13039/501100005915Tata Trusts grant number Health-BSDT-20151007.

## Author contribution

SC: Conceptualization, Methodology, Data Analysis, Visualization, Writing – review & editing. SB: Data analysis, Visualization, Writing – original draft. BK, TW: Methodology, Investigation, Data Analysis. VG: Methodology, Visualization, Validation, Writing – review & editing. VD: Conceptualization, Methodology, Supervision, Writing – review & editing. SS: Conceptualization, Methodology, Supervision, Resources. SPS: Conceptualization, Methodology, Supervision, Resources, Writing – review & editing.

## Declaration of competing interest

The authors declare no conflict of interest.
