# Supplementary material for: Lipid-based ayurvedic formulations of a single herb-Yashtimadhu (Glycyrrhiza glabra): Pharmaceutical standardization, shelf-life estimation and comparative characterization
Source: J Ayurveda Integr Med. 2023 Apr 25;14(2):100711. doi: 10.1016/j.jaim.2023.100711 (PMC10164906; doi:10.1016/j.jaim.2023.100711)
Supplement: Multimedia component 1 [file mmc1.docx]

**Supplementary file**

**Table S1:** Monograph of *Yashtimadhu Ghrut* (YG) and *Yashtimadhu Taila* (YT)

| Parameters | Unit | | YG | YT |
| --- | --- | --- | --- | --- |
| Physicochemical parameters | |  | | |
| Description |  | | Yellow coloured thick ghee (liquid) | Yellow coloured thick viscous oil (liquid) |
| Specific gravity | -- | | 0.90-0.91 | 0.90-0.91 |
| Loss on drying at 105 °C | % | | Not More Than 0.5 | Not More Than 0.1 |
| Acid value | -- | | Not More Than 2 | Not More Than 2 |
| Refractive Index | -- | | 1.53-1.54 | 1.53-1.54 |
| Saponification Value | -- | | 185-250 | 170-250 |
| Iodine value | -- | | 20-35 | 30-80 |
| Peroxide value | -- | | Not More Than 2 | Not More Than 1 |
| Unsaponifiable matter | -- | | Not More Than 1 | Not More Than 1.5 |
| Reichert Meissl value | -- | | 30-45 | NA |
| Cotton seed oil | -- | | NA | Absent |
| Congealing point | ^0^C | | 19-28 | NA |
| Safety parameters | | | | |
| Heavy metals (As, Cd, Pb, Hg) |  | | Within permissible limit | Within permissible limit |
| Microbial count | (cfu/g) | | NMT 10^5^ | NMT 10^5^ |
| Fungal count | (cfu/g) | | NMT 10^3^ | NMT 10^3^ |
| *Escherichia coli* | (cfu/g) | | Absent | Absent |
| Thin Layer Chromatography | | | | |
| 256 nm | *R_f_* (color) | | 1 spot: 0.95(B) | 13 spots: 0.48, 0.62, 0.63, 0.65 (B), 0.67, 0.68(Gr), 0.69(Y), 0.71(Gr), 0.72, 0.77, 0.82, 0.86, 0.94 (B) |
| 360 nm | *R_f_* (color) | | 4 spots: 0.49, 0.57, 0.59 (LB), 0.64(R) | 10 spots: 0.43, 0.52, 0.56 (B), 0.62(B), 0.63, 0.67, 0.69, 0.71, 0.76(R), 0.77 (B) |
| Anisaldehyde sulphuric acid reagent | *R_f_* (color) | | 11 spots: 0.54, 0.57(B), 0.59(R), 0.66, 0.69, 0.70, 0.75, 0.79, 0.80, 0.82, 0.95 (B) | 14 spots: 0.27, 0.29(B), 0.43 (P), 0.55(Gr), 0.57(V), 0.60 (O), 0.67, 0.71 (B), 0.72 (Y), 0.76(PV), 0.77(P), 0.81(B), 0.87, 0.96 (Br) |
| High Performance Liquid Chromatography | | | | |
| Glabridin | (g/100mL) | | 0.009 (95 ppm) | 0.014 (138 ppm) |
| 18-ß Glycyrrhetinic acid | (g/100mL) | | 0.011 (112.3 ppm) | 0.05 (500 ppm) |
| Stability study (shelf-Life analysis) | | | | |
| Climatic Zone (I & II) | Months | | 20.83 | 8.03 |
| Climatic zone (III & IV) | Months | | 31.57 | 12.16 |

NA: Not applicable B= Blue, Br= Brown, R= Red, Gr=Green, Gy= Grey, V=Violet, PV=Pink Violet, Bl=Black, Flu B=Fluorescent Blue, Y= Yellow, P=Pink, O= Orange, LB= Light Blue

**Table S2**

Heavy metal analysis of three batches of YG and YT by ICP-OES

|  | Steps | As | | Cd | | Hg | | Pb | |
| --- | --- | --- | --- | --- | --- | --- | --- | --- | --- |
| YG |  | **Batch 1** | | | | | | | |
|  | Weight (g) | 0.5369 | 0.5179 | 0.5369 | 0.5179 | 0.5369 | 0.5179 | 0.5369 | 0.5179 |
|  | Reading in Conc. | -1.063 | -1.291 | 0.299 | 0.219 | 1.276 | 1.303 | 3.249 | 2.445 |
|  | Result | -0.099 | -0.125 | 0.028 | 0.021 | 0.119 | 0.126 | 0.303 | 0.236 |
|  | Mean | -0.112 | | 0.024 | | 0.122 | | 0.269 | |
|  |  | **Batch 2** | | | | | | | |
|  | Weight (g) | 0.5739 | 0.5325 | 0.5739 | 0.5325 | 0.5739 | 0.5325 | 0.5739 | 0.5325 |
|  | Reading in Conc. | -0.691 | -1.613 | 0.223 | 0.326 | 0.355 | 0.716 | 2.257 | 4.302 |
|  | Result | -0.060 | -0.151 | 0.019 | 0.031 | 0.031 | 0.067 | 0.197 | 0.404 |
|  | Mean | -0.106 | | 0.025 | | 0.049 | | 0.300 | |
|  |  | **Batch 3** | | | | | | | |
|  | Weight (g) | 0.5082 | 0.5120 | 0.5082 | 0.5120 | 0.5082 | 0.5120 | 0.5082 | 0.5120 |
|  | Reading in Conc. | -0.645 | -1.838 | 0.243 | 0.185 | 0.58 | 0.22 | 4.326 | 2.746 |
|  | Result | -0.063 | -0.179 | 0.024 | 0.018 | 0.057 | 0.021 | 0.426 | 0.270 |
|  | Mean | -0.121 | | 0.021 | | 0.039 | | 0.348 | |
|  |  | **Batch 1** | | | | | | | |
| YT | Weight (g) | 0.5008 | 0.5002 | 0.5008 | 0.5002 | 0.5008 | 0.5002 | 0.5008 | 0.5002 |
|  | Reading in Conc. | -0.581 | -0.688 | -0.027 | -0.073 | -3.258 | -6.420 | 0.317 | 1.944 |
|  | Result | -0.058 | -0.069 | -0.003 | -0.007 | -0.325 | -0.642 | 0.032 | 0.194 |
|  | Mean | -0.063 | | -0.005 | | -0.484 | | 0.113 | |
|  |  | **Batch 2** | | | | | | | |
|  | Weight (g) | 0.5004 | 0.5011 | 0.5004 | 0.5011 | 0.5004 | 0.5011 | 0.5004 | 0.5011 |
|  | Reading in Conc. | 0.293 | -0.853 | 0.098 | -0.097 | -9.039 | -7.202 | 0.262 | 0.811 |
|  | Result | 0.029 | -0.085 | 0.010 | -0.010 | -0.903 | -0.719 | 0.026 | 0.081 |
|  | Mean | -0.028 | | 0.000 | | -0.811 | | 0.054 | |
|  |  | **Batch 3** | | | | | | | |
|  | Weight (g) | 0.5008 | 0.5001 | 0.5008 | 0.5001 | 0.5008 | 0.5001 | 0.5008 | 0.5001 |
|  | Reading in Conc. | 1.286 | 2.192 | -0.019 | -0.299 | 7.383 | 6.253 | 0.772 | 0.323 |
|  | Result | 0.128 | 0.219 | -0.002 | -0.030 | 0.737 | 0.625 | 0.077 | 0.032 |
|  | Mean | 0.174 | | -0.016 | | 0.681 | | 0.055 | |

All mean values as mg/L

**Table S3**

Microbial load analysis of finished products

|  | Microbial count (cfu/gm) | | | Fungal count (cfu/gm) | | | *E. coli* | | |
| --- | --- | --- | --- | --- | --- | --- | --- | --- | --- |
|  | Batch 1 | Batch 2 | Batch 3 | Batch 1 | Batch 2 | Batch 3 | Batch 1 | Batch 2 | Batch 3 |
| YG | 10000 | 300 | 600 | Nil | Nil | Nil | Absent | Absent | Absent |
| YT | 2x10^4^ | Nil | 1000 | 3x10^3^ | Nil | 1000 | Absent | Absent | Absent |

**Table S4**

Accelerated and Long-term study of YG

|  | 0 Day | | 3 Months | | 6 Months | | 9 months | 12 Months |
| --- | --- | --- | --- | --- | --- | --- | --- | --- |
|  | LT | AS | LT | AS | LT | AS |  |  |
| Description | Yellow thick sticky semisolid ghrut (mass) having characteristic odour | | | | | | | |
| Specific gravity | 0.91±0.00 | 0.91±0.00 | 0.92±0.00 | 0.91±0.00 | 0.91±0.00 | 0.91±0.00 | 0.91±0.00 | 0.92±0.00 |
| Refractive index | 1.53±0.00 | 1.53±0.00 | 1.53±0.00 | 1.53±0.00 | 1.53±0.00 | 1.53±0.00 | 1.53±0.00 | 1.53±0.00 |
| Acid value | 1.94±0.10 | 1.94±0.10 | 2.39±0.14 | 2.96±0.66 | 2.53±0.12 | 3.58±0.28 | 3.58±0.61 | 4.68±1.46 |
| Saponification value | 243.09±1.21 | 243.09±1.21 | 226.81±9.49 | 246.17±3.20 | 223.71±11.26 | 229.10±10.77 | 223±3.75 | 162.72±13.08 |
| Iodine value | 40.53±5.97 | 40.53±5.97 | 16.56±4.07 | 18.97±2.62 | 38.87±2.82 | 27.20±0.47 | 25.69±2.12 | 26.36±1.38 |
| Peroxide value | 1.21±0.26 | 1.21±0.26 | 1.83±0.30 | 1.31±0.27 | 2.32±0.61 | 1.78±0.16 | 2.28±0.47 | 2.15±1.27 |
| Unsaponifiable matter | 3.13±0.57 | 3.13±0.57 | 8.07±0.95 | 4.36±0.08 | 4.21±0.44 | 3.35±0.43 | 4.83±0.66 | 4.37±0.82 |
| Loss on drying | 0.24±0.14 | 0.24±0.14 | 0.16±0.06 | 0.31±0.22 | 0.15±0.02 | 1.91±0.31 | 0.19±0.02 | 0.83±0.22 |
| Congealing point | 17±0.58 | 17±0.58 | 17.33±0.33 | 14.67±0.88 | 16.67±0.88 | 17±0.58 | 14.67±0.33 | 13.33±0.33 |
| Heavy metals | 20±0.00 | 20±0.00 | 20±0.00 | 20±0.00 | 20±0.00 | 20±0.00 | 20±0.00 | 20±0.00 |
| Microbial count | NMT 10^4^ | NMT 10^4^ | NMT 7x10^3^ | NMT 10^3^ | NMT 8x10^4^ | NMT 8x 10^5^ | NMT 10^5^ | NMT 3x 10^4^ |
| Fungal count | NMT 2x10^3^ | NMT 2x10^3^ | NMT 200 | NMT 3x10^3^ | NMT 5x10^3^ | NMT 6x10^4^ | NMT 2x10^4^ | NMT 10^4^ |
| *E Coli* (CFU/g) | Absent | Absent | Absent | Absent | Absent | Absent | Absent | Absent |
| Thin Layer chromatography (no. of spots) | | | | | | | | |
| 256nm | 8 | 8 | 8 | 11 | 9 | 11 | 10 | 7 |
| 360nm | 9 | 10 | 10 | 11 | 12 | 14 | 14 | 11 |
| Anisaldehyde sulphuric acid reagent | 11 | 10 | 10 | 12 | 15 | 13 | 12 | 11 |
| HPLC | | | | | | | | |
| Glabridin (g/100mL) | 0.01 | 0.01 | 0.008 | 0.007 | 0.006 | 0.007 | 0.009 | 0.014 |
| 18-ß Glycyrrhetinic acid (g/100mL) | 0.01 | 0.01 | 0.004 | 0.004 | 0.003 | 0.003 | 0.002 | 0.002 |

NMT: Not More Than

**Table S5**

Accelerated and Long-term study of YT

|  | 0 Day | | 3 Months | | 6 Months | | 9 months | 12 Months |
| --- | --- | --- | --- | --- | --- | --- | --- | --- |
|  | LT | AS | LT | AS | LT | AS |  |  |
| Description | Yellow colored thick viscous (liquid) oil having characteristic odor | | | | | | | |
| Specific gravity | 0.92±0.00 | 0.92±0.00 | 0.92±0.00 | 0.91±0.00 | 0.92±0.00 | 0.92±0.00 | 0.92±0.00 | 0.92±0.00 |
| Refractive index | 1.54±0.00 | 1.54±0.00 | 1.54±0.00 | 1.54±0.00 | 1.54±0.00 | 1.54±0.00 | 1.54±0.00 | 1.54±0.00 |
| Acid value | 2.40±0.00 | 2.40±0.00 | 3.26±0.00 | 2.30±0.05 | 3.19±0.00 | 3.21±0.10 | 3.06±0.09 | 2.85±0.18 |
| Saponification value | 225.33±13.99 | 225.33±13.99 | 225.57±4.26 | 219.31±5.45 | 199.73±12.43 | 231.54±13.08 | 194.02±5.69 | 222.16±5.87 |
| Iodine value | 120.28±2.25 | 120.28±2.25 | 70.55±12.24 | 41±3.40 | 19.71±10.3 | 27.21±3.84 | 46.73±12.26 | 41.82±0.34 |
| Peroxide value | 2.25±0.55 | 2.25±0.55 | 0.29±0.06 | 3.25±0.72 | 2.42±0.56 | 1.41±0.08 | 3.53±1.06 | 3.58±0.43 |
| Unsaponifiable matter | 2.59±0.88 | 2.59±0.88 | 4.12±0.26 | 4.82±0.85 | 5.83±0.71 | 5.92±0.93 | 5.53±1.20 | 18.50±7.55 |
| Loss on drying | 0.12±0.05 | 0.12±0.05 | 0.20±0.03 | 0.11±0.03 | 0.6±0.1 | 0.14±0.00 | 0.16±0.02 | 0.20±0.04 |
| Heavy metals | 20.00±0.00 | 20.00±0.00 | 20.00±0.00 | 20.00±0.00 | 20.00±0.00 | 20.00±0.00 | 20.00±0.00 | 20±0.00 |
| Microbial count | NMT 3x10^3^ | NMT 3x10^3^ | NMT 2x10^4^ | NMT 2x10^5^ | NMT 10^4^ | NMT 10^4^ | NMT 10^5^ | NMT 2x10^5^ |
| Fungal count | Absent | Absent | NMT 4x10^4^ | Absent | NMT 600 | NMT 10^4^ | NMT 3x10^3^ | NMT 4x10^4^ |
| *E Coli* (CFU/g) | Absent | Absent | Absent | Absent | Absent | Absent | Absent | Absent |
| Thin Layer chromatography (No. of spots) | | | | | | | | |
| 256nm | 9 | 9 | 9 | 7 | 15 | 10 | 15 | 12 |
| 360nm | 9 | 9 | 9 | 12 | 15 | 12 | 14 | 14 |
| Anisaldehyde sulphuric acid reagent | 12 | 8 | 10 | 9 | 14 | 11 | 16 | 14 |
| HPLC | | | | | | | | |
| Glabridin (g/100mL) | 0.05 | 0.05 | 0.036 | 0.034 | 0.0035 | 0.034 | 0.047 | 0.061 |
| 18-ß Glycyrrhetinic acid (g/100mL) | 0.004 | 0.004 | 0.003 | 0.003 | 0.002 | 0.002 | 0.002 | 0.001 |

NMT: Not More Than

**Table S6**

Details of TLC study in accelerated and long-term study of YG

|  | 0 Day | | 3 Months | | 6 Months | | 9 months | 12 Months |
| --- | --- | --- | --- | --- | --- | --- | --- | --- |
|  | LT | AS | LT | AS | LT | AS |  |  |
| 256nm | 0.79, 0.866 (GB), 0.915, 0.951 (B), 0.925 (Y), 0.94 (G), 0.983 (B), 0.991(Y) | 0.79, 0.805 (B), 0.85 (G), 0.915, 0.925 (Y), 0.94 (G), 0.983 (B), 0.991 (Y) | 0.357, 0.41, 0.44, 0.473, 0.57, 0.63 (Y), 0.74 (GB), 0.79 (B), | 0.596, 0.605, 0.637, 0.74(B), 0.817(Y), 0.823 (Y), 0.836 (B), 0.897(Y), 0.902 (GY), 0.932 (B), 0.946(GY), | 0.504, 0.626, 0.684 (B), 0.721 (Y), 0.763 (B), 0.808, 0.829 (V), 0.845 (Y), 0.878(OY) | 0.516, 0.525, 0.558, 0.633 (Y), 0.666 (VB), 0.683(Y), 0.725 (V), 0.733 (VB), 0.766, 0.833, 0.851(OY) | 0.527, 0.546, 0.563, 0.583, 0.592, 0.611, 0.712, 0.757(Y), 0.814, 0.825(B) | 0.59, 0.66 (Y), 0.72 (GY), 0.77 (Y), 0.79 (GY), 0.83 (Y), 0.88 (GY) |
| 360nm | 0.799 (B), 0.805, 0.844 (GB), 0.858 (BV), 0.866 (B), 0.91 (BV), 0.941 (V), 0.951 (GB), 0.975 (O) | 0.799(B), 0.805 (GB),0.844 (GB), 0.858 (BV), 0.866 (B), 0.91(BV), 0.941 (V), 0.951 (GB), 0.975 (O), 0.983 (OY) | 0.43(Y), 0.45(B), 0.56, 0.58(Y), 0.63(O), 0.65(R), 0.663(Y), 0.68(O), 0.705 (Y), 0.78 (R) | 0.586, 0.605, 0.625, 0.634, 0.646, 0.681 (B), 0.692, 0.725(Y), 0.883(RP), 0.902 (GB), 0.938 (Y) | 0.608, 0.634 (Y), 0.666 (B), 0.692(FB), 0.730 (GrY), 0.736 (FB), 0.773 (GrY), 0.808 (OY), 0.826, 0.850 (OV), 0.884 (OY), 0.886 (Y) | 0.508 (B), 0.608 (G), 0.625 (Y), 0.633, 0.683 (B), 0.691 (Y), 0.733 (B), 0.741 (Y), 0.775 (Y), 0.808, 0.841 (B), 0.853 (Y), 0.875, 0.891 (V) | 0.537, 0.564, 0.583, 0.66 (Y), 0.675 (BV), 0.712, 0.777 (Y), 0.805 (BV),  0.825 (GB), 0.842 (B), 0.851(V), 0.864 (GB), 0.886 (V), 0.902(B) | 0.58, 0.60, 0.64, 0.68 (B), 0.74 (GY), 0.75 (FB), 0.78(GY), 0.83, 0.87, 0.89(O), 0.91(Y) |
| Anisaldehyde sulphuric acid reagent | 0.417, 0.583, 0.766, 0.75 (B), 0.796 (RP), 0.81(B), 0.858(V), 0.883(RP), 0.89, 0.958, 0.96(O) | 0.417, 0.583, 0.766, 0.75(B), 0.796 (RP), 0.81, 0.858 (V), 0.883 (RP), 0.89, 0.96 (O) | 0.389, 0.43, 0.505, 0.52(Y), 0.55 (R), 0.57(Y), 0.61(PO), 0.65(B), 0.68(PO), 0.78 (B) | 0.477, 0.49 (B), 0.567, 0.625 (GB), 0.653 (B), 0.701, 0.79(Y), 0.80 (OP), 0.814 (Y), 0.858 (RP), 0.885, 0.894(RB) | 0.46, 0.556 (V), 0.626(OB), 0.634(B), 0.704(OB), 0.728(Y), 0.763(VO), 0.773(Y), 0.808(O), 0.815(BrO), 0.817, 0.852(OY), 0.859, 0.878, 0.93(V) | 0.466, 0.508 (O), 0.6, 0.616 (BO), 0.633 (O), 0.666 (G), 0.675 (GO), 0.70, 0.725 (BG), 0.75, 0.783, 0.80, 0.85(V) | 0.514, 0.527(Y), 0.537, 0.583(OY), 0.65(Y), 0.703, 0.708, 0.771(O), 0.814(V), 0.824, 0.861(GB), 0.893(B) | 0.57, 0.65, 0.67, 0.72(O), 0.75, 0.79(G), 0.83(B), 0.87(O), 0.94(V), 0.96(O), 0.97(B) |

B= Blue, G=Green, Y= Yellow, GY= Green yellow, V=violet, O= Orange, VB=Violet blue, BV= blue violet, GB= Greenish blue, FB= florescent blue, OV=orange violet, OY=Orange yellow, PO=Pink orange, OB=Orange blue, OP= Orange pink, VO= Violet orange, BrO= brownish orange, RP= Red pink, RB= Red blue, BG= Blue green, BO= blue orange, GO= green orange

**Table S7**

Details of TLC study in accelerated and long-term study of YT

|  | 0 Day | | 3 Months | | 6 Months | | 9 months | 12 Months |
| --- | --- | --- | --- | --- | --- | --- | --- | --- |
|  | LT | AS | LT | AS | LT | AS |  |  |
| 256nm | 0.575, 0.624, 0.654, 0.71, 0.7454, 0.80, 0.84, 0.86, 0.92 (B) | 0.575, 0.624, 0.654, 0.71, 0.7454, 0.80, 0.84, 0.866, 0.92 (B) | 0.476, 0.552, 0.649, 0.738, 0.742, 0. 761, 0.89, 0.923, 0.971 (B) | 0.77 (B), 0.820 (Y), 0.836 (B), 0.844 (Y), 0.854, 0.868, 0.911(B) | 0.570 (Y), 0.557, 0.585(B), 0.59, 0.668, 0.678, 0.689, 0.696 (Y), 0.709(B), 0.717(Y), 0.75, 0.79 (DB), 0.812, 0.840, 0.865 (P) | 0.53 (B), 0.591 (Y), 0.633 (GB), 0.683 (OY), 0.725, 0.783 (Y), 0.816, 0.858 (YB), 0.883 (OP), 0.975 (O) | 0.495, 0.469, 0.547(B), 0.557(GB), 0.595, 0.626(B), 0.654, 0.658(GB), 0.672 (YBr), 0.769(YP), 0.803 (YBr), 0.814(YO), 0.857(YP), 0.878, 0.943(OY) | 0.58, 0.613, 0.62, 0.73, 0.754(Y), 0.78(OY), 0.8, 0.81(Y), 0.852 (OY),0.911, 0.92, 0. 96(Y) |
| 360nm | 0.6, 0.766, 0.773, 0.73, 0.7454, 0.853, 0.893, 0.866, 0.933(B) | 0.6, 0.73, 0.7454, 0.773, 0.766, 0.853, 0.866, 0.893,0.933 (B) | 0.447, 0.559, 0.611, 0.695, 0.64, 0.716(B), 0.809, 0.819 (OY), 0.971(O) | 0.187, 0.196, 0.671, 0.696(B), 0.757, 0.796 (Y), 0.806 (B), 0.844 (B), 0.870 (Y), 0.906 (O), 0.935, 0.967 (B) | 0.527 (GB), 0.58 (Y), 0.565 (GB), 0.579, 0.621(B), 0.68, 0.709, 0.73(Y), 0.745, 0.773, 0.809(B), 0.817(P), 0.835 (Y), 0.846, 0.878(P) | 0.571, 0.622 (Y), 0.704 (OY), 0.733 (B), 0.708 (Y), 0.764, 0.795, 0.808 (OY), 0.841 (O), 0.858 (Y), 0.911 (OP), 0.958 (Y) | 0.486, 0.50 0.504, 0.537 (B), 0.611, 0.637(Y), 0.644, 0.663(BG), 0.694 (Y),0.777(YO), 0.787(P), 0.850, 0.896, 0.962(OY) | 0.57, 0.588, 0.64 (B), 0.656, 0.72, 0.725(Y), 0.75(V), 0.803, 0.83, 0.88(OY), 0.901 (V), 0. 92(V), 0.94(OY), 0.97 (Y) |
| Anisaldehyde sulphuric acid reagent | 0.40, 0.446, 0.46 (P),0.51, 0.55, 0.71, 0.7151(O), 0.873(V), 0.88(P), 0.904(V), 0.933(O), 0.98(V) | 0.4, 0.46(P), 0.55, 0.715 (O), 0.873 (V), 0.88 (P), 0.904, 0.98(V) | 0.38, 0.383(B), 0.485(P), 0.609(B), 0.611, 0.708, 0.790(O), 0.809(B), 0.923(PO), 0.980(O) | 0.726, 0.741 (B), 0.789 (O), 0.79 (OY), 0.828, 0.844, 0.885, 0.898 (O), 0.926 (V) | 0.576, 0.587, 0.597(P), 0.625, 0.630(G), 0.634, 0.678, 0.695(P), 0.736, 0.751(OP), 0.804, 0.824(GBr), 0.853, 0.901(GB) | 0.466 (B), 0.54 (V), 0.641, 0.65 (B), 0.675 (Y), 0.691 (V), 0.724 (B), 0.775 (VB), 0.826 (OV), 0.841 (V), 0.875 (OP) | 0.485, 0.495(B), 0.515(Y), 0.557(GB), 0.57, 0.588 (Y), 0.601, 0.635(Y), 0.663(YP), 0.682(GP), 0.698(Y), 0.786(YP), 0.794(PO), 0.831(OP), 0.928(Y), 0.915(O) | 0.52, 0.55, 0.568(B) 0.617, 0.63, 0.67(Y), 0.74(GY), 0.75, 0.794(OY), 0.87(GY), 0.88(V), 0.911(OY), 0.92, 0.96(V) |

B= blue, Y= Yellow, O= Orange, P= Pink, V=violet, GB= Greenish blue, YB= Yellowish blue, OP= Orange pink, OV= Orange violet, VB= Violet blue, BY=brownish yellow, OV= orange violet, VB= violet blue, OY= Orange yellow, YBr= Yellow brown, YO= Yellow orange, YP= Yellow pink, DB= Dark blue, GBr= Greenish brown, OP= Orange pink, PO=Pink orange, GP= Greenish pink, GY= Green yellow

**Table S8**

Degradation percentage and theoretical range for physicochemical characters of YG at accelerated and long-term storage condition

|  |  | LT | | | | AS | | | |  |  |
| --- | --- | --- | --- | --- | --- | --- | --- | --- | --- | --- | --- |
| Parameter | Initial | Degradation % | | Range | | Degradation % | | Range | | Final  (6 months) | Final  (12 months) |
|  |  | **15%** | **25%** | ±**15%** | ±**25%** | **15%** | **25%** | ±**15%** | ±**25%** |  |  |
| Acid value | 1.94 | ±0.29 | ±0.49 | 1.65-2.23 | 1.46-2.43 | ±0.29 | ±0.49 | 1.65-2.23 | 1.46-2.43 | 3.58 | 4.68 |
| Peroxide value | 1.21 | ±0.18 | ±0.30 | 1.03-1.39 | 0.91-1.51 | ±0.18 | ±0.30 | 1.03-1.39 | 0.91-1.51 | 1.78 | 2.15 |
| Iodine value | 40.53 | ±6.08 | ±10.13 | 34.45-46.61 | 30.40-50.67 | ±6.08 | ±10.13 | 34.45-46.61 | 30.40-50.66 | 27.20 | 26.36 |
| Saponification value | 243.09 | ±36.46 | ±60.77 | 206.63-  279.55 | 182.32-303.86 | ±36.46 | ±60.77 | 206.63-279.55 | 182.32-303.86 | 229.10 | 162.72 |
| Unsaponifiable matter | 3.13 | ±0.47 | ±0.78 | 2.66-3.60 | 2.35-3.91 | ±0.47 | ±0.78 | 2.66-3.60 | 2.35-3.91 | 3.35 | 4.37 |
| Loss on drying | 0.24 | ±0.04 | ±0.06 | 0.21-0.28 | 0.18-0.31 | ±0.04 | ±0.06 | 0.21-0.28 | 0.18-0.3 | 1.91 | 0.83 |
| Congealing point | 17 | ±2.55 | ±4.25 | 14.45-19.55 | 12.75-21.25 | ±2.55 | ±4.25 | 14.45-19.55 | 12.75-21.25 | 17.00 | 13.33 |
| Specific gravity | 0.91 | ±0.14 | ±0.23 | 0.77-1.05 | 0.68-1.14 | ±0.14 | ±0.23 | 0.77-1.05 | 0.68-1.14 | 0.91 | 0.92 |
| Refractive index | 1.53 | ±0.23 | ±0.38 | 1.30-1.76 | 1.15-1.92 | ±0.23 | ±0.38 | 1.30-1.76 | 1.15-1.91 | 1.53 | 1.53 |
| Glabridin | 0.011 | ±0.00165 | ±0.00275 | 0.009-  0.0129 | 0.008-0.0140 | ±0.0017 | ±0.0028 | 0.0095-0.0129 | 0.0014-0.0084 | 0.0065333 | 0.014 |
| 18-ß Glycyrrhetinic acid | 0.01 | ±0.0015 | ±0.0025 | 0.009-  0.0109 | 0.008-0.0119 | ±0.0014 | ±0.0024 | 0.0081-  0.0109 | 0.0071-0.0119 | 0.0026 | 0.002 |

**Table S9**

Degradation percentage and theoretical range for physicochemical characters of YT at long-term and accelerated storage condition

|  |  | LT | | | | AS | | | |  |  |
| --- | --- | --- | --- | --- | --- | --- | --- | --- | --- | --- | --- |
| Parameter | Initial | Degradation % | | Range | | Degradation % | | Range | | Final  (6 months) | Final  (12 months) |
|  |  | **25%** | **15%** | **±15%** | **±25%** |  |  |  |  |  |  |
| Acid value | 2.40 | ±0.60 | ±0.36 | 2.04-2.76 | 1.80-3 | ±0.36 | ±0.60 | 2.037-2.756 | 1.8-2.996 | 3.207 | 2.85 |
| Iodine value | 120.28 | ±30.07 | ±18.04 | 102.24-138.33 | 90.21-150.36 | ±18.04 | ±30.07 | 102.242-  138.33 | 90.21-150.35 | 27.211 | 41.82 |
| Peroxide value | 2.25 | ±0.56 | ±0.34 | 1.91-2.58 | 1.68-2.81 | ±0.34 | ±0.56 | 1.909-2.586 | 1.68-2.806 | 1.405 | 3.58 |
| Saponification value | 225.33 | ±56.33 | ±33.80 | 191.53-259.13 | 169-281.67 | ±33.80 | ±56.33 | 191.53-259.13 | 169-281.66 | 231.54 | 222.16 |
| Unsaponifiable matter | 2.59 | ±0.65 | ±0.39 | 2.2-2.98 | 1.94-3.24 | ±0.39 | ±0.65 | 2.204-2.983 | 1.94-3.243 | 5.920 | 18.50 |
| Loss on drying | 0.12 | ±0.03 | ±0.02 | 0.1-0.14 | 0.09-0.15 | ±0.02 | ±0.03 | 0.102-0.14 | 0.09-0.15 | 0.135 | 0.20 |
| Specific gravity | 0.92 | ±0.23 | ±0.14 | 0.78-1.05 | 0.69-1.15 | ±0.14 | ±0.23 | 0.779-1.056 | 0.69-1.146 | 0.917 | 0.92 |
| Refractive index | 1.54 | ±0.39 | ±0.23 | 1.31-1.77 | 1.16-1.93 | ±0.23 | ±0.39 | 1.31-1.772 | 1.16-1.932 | 1.541 | 1.54 |
| Glabridin | 0.0500 | ±0.01 | ±0.0075 | 0.0425-0.058 | 0.04-0.063 | ±0.0075 | ±0.0125 | 0.043-  0.0575 | 0.038-0.0625 | 0.033833 | 0.0613 |
| 18-ß Glycyrrhetinic acid | 0.0038 | ±0.0010 | ±0.00057 | 0.0032-0.0044 | 0.0029-0.0048 | ±0.00057 | ±0.00095 | 0.0032-  0.0044 | 0.0029-0.0048 | 0.0017 | 0.0014 |


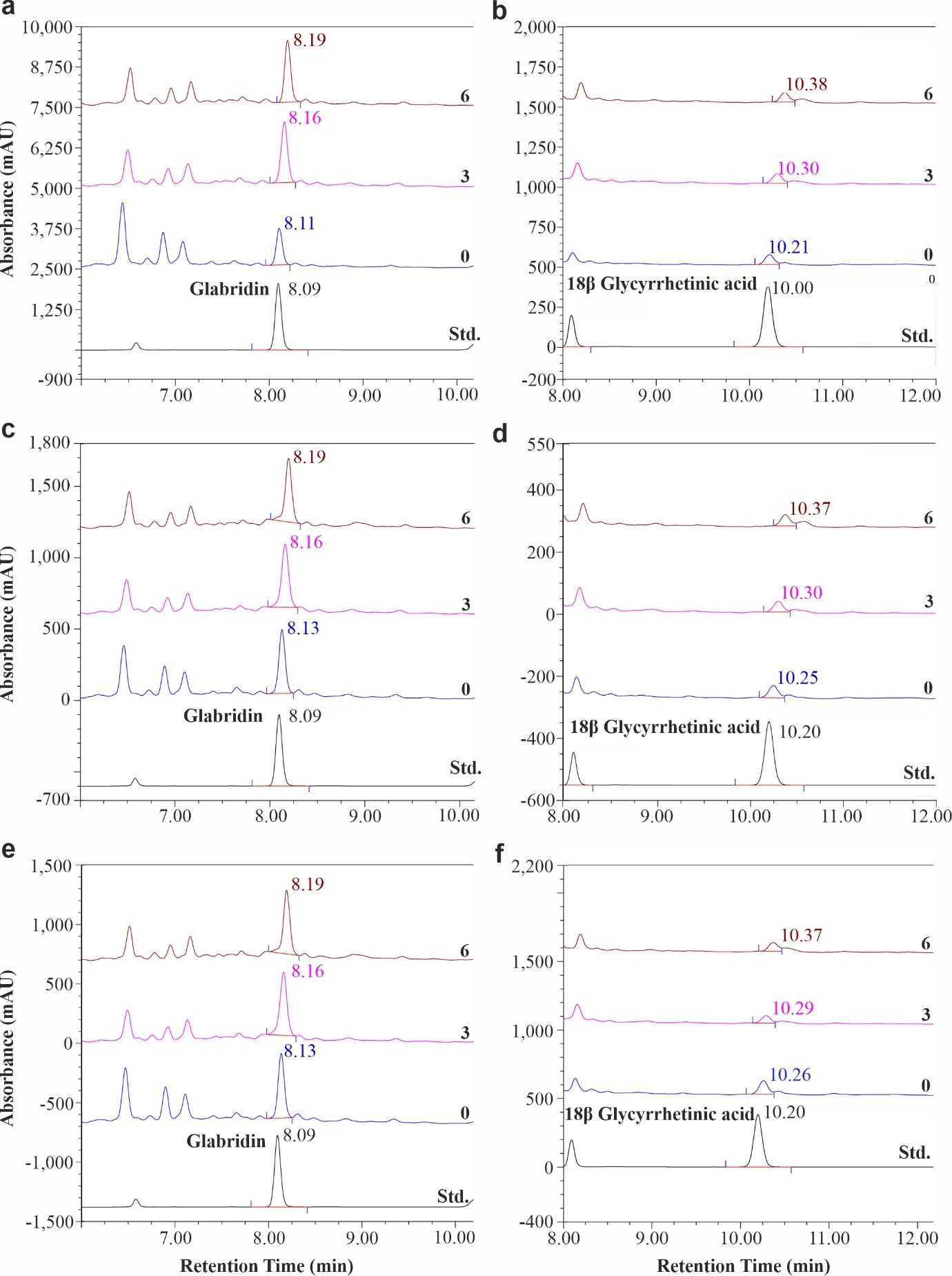


**Fig.S1.** HPLC chromatograms of three batches of YG showing the presence of glabridin (at 230 nm) and 18ß glycyrrhetinic acid (at 254 nm) at 0, 3, and 6-month time points in accelerated stability study (a,b) Batch YG1 (c,d) Batch YG2 and (e,f) Batch YG3.


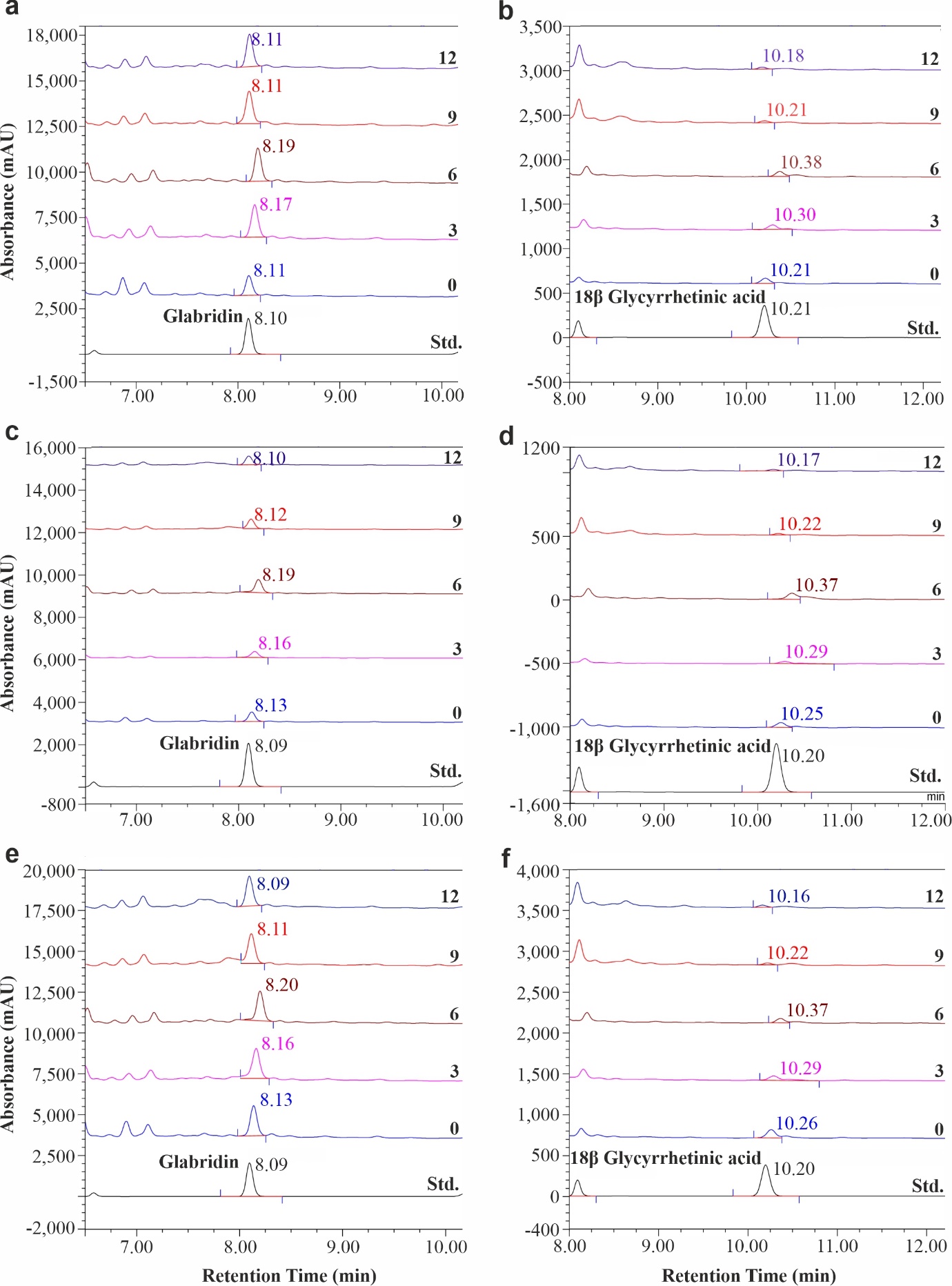


**Fig.S2.** HPLC chromatograms of three batches of YG showing the presence of glabridin (at 230 nm) and 18ß glycyrrhetinic acid (at 254 nm) at 0, 3, 6, 9, and 12-month time points in long-term stability study (a,b) Batch YG1 (c,d) Batch YG2 and (e,f) Batch YG3.


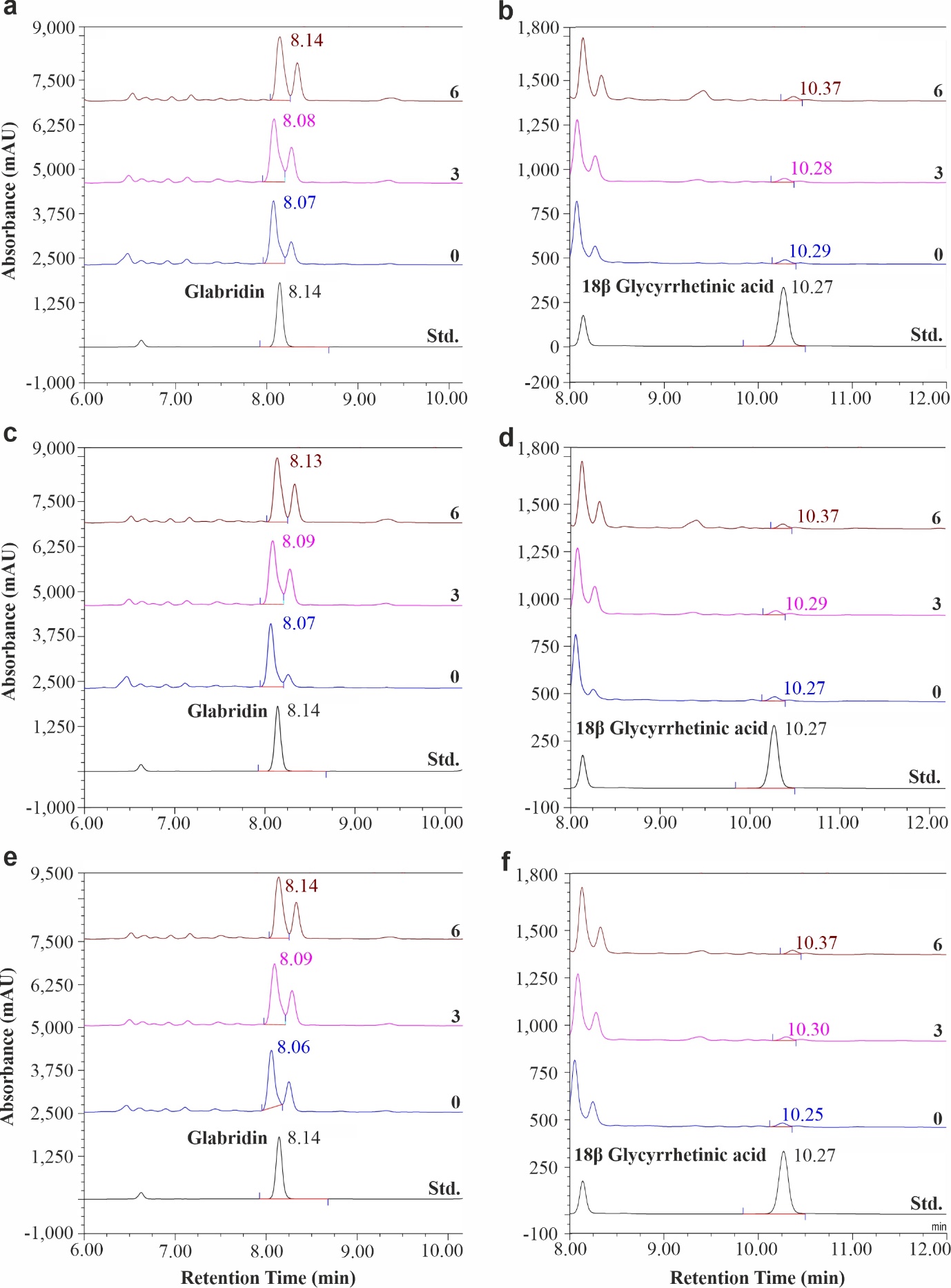


**Fig.S3.** HPLC chromatograms of three batches of YT showing the presence of glabridin (at 230 nm) and 18ß glycyrrhetinic acid (at 254 nm) at 0, 3, and 6-month time points in accelerated stability study (a,b) Batch YT1 (c,d) Batch YT2 and (e,f) Batch YT3.


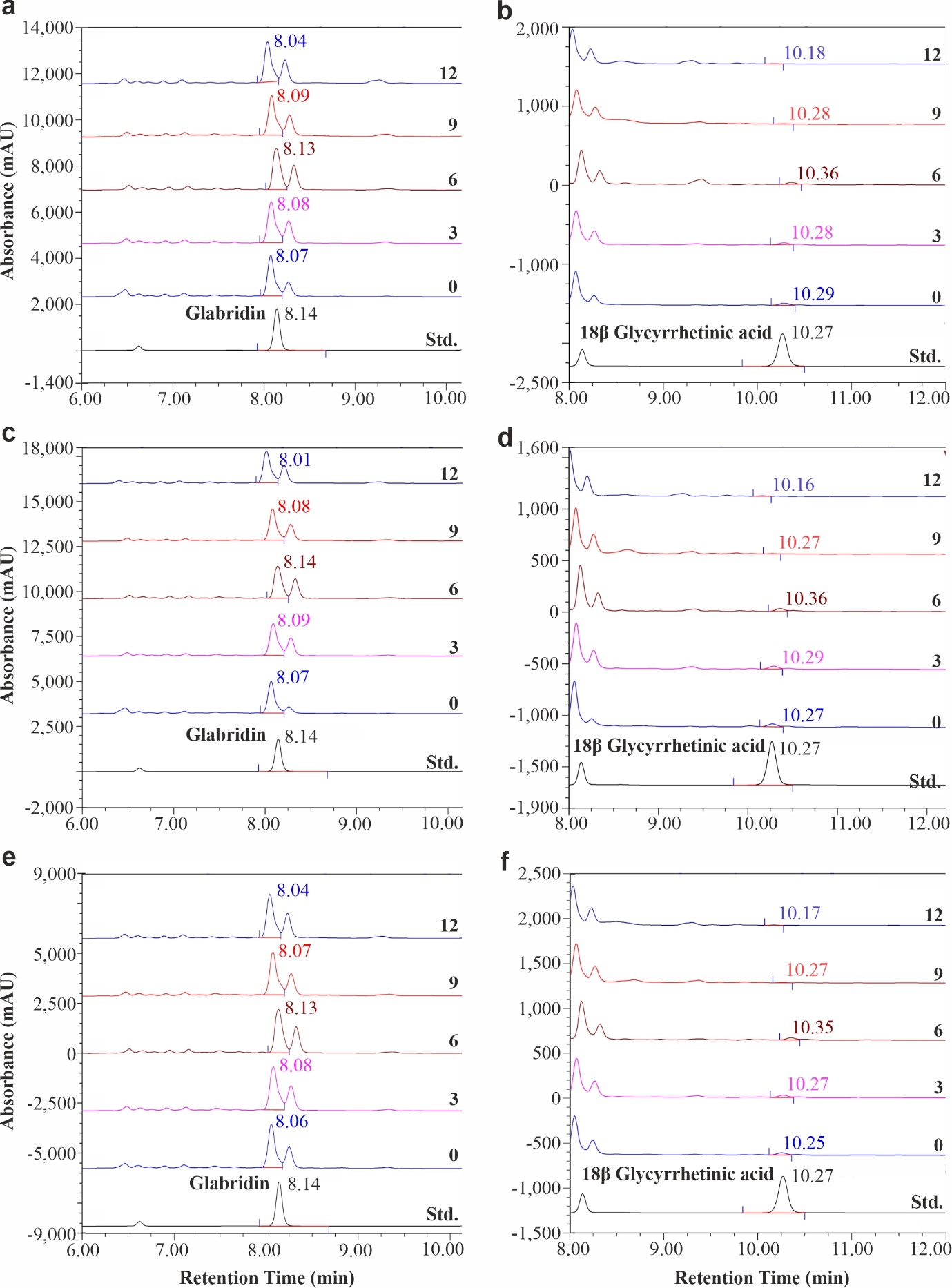


**Fig.S4.** HPLC chromatograms of three batches of YT showing the presence of glabridin (at 230 nm) and 18ß glycyrrhetinic acid (at 254 nm) at 0, 3, 6, 9, and 12-month time points in long-term stability study (a,b) Batch YT1 (c,d) Batch YT2 and (e,f) Batch YT3.
